# Ultrasound-Assisted Extraction, LC–MS/MS Analysis, Anticholinesterase, and Antioxidant Activities of Valuable Natural Metabolites from *Astragalus armatus* Willd.: In Silico Molecular Docking and In Vitro Enzymatic Studies

**DOI:** 10.3390/antiox11102000

**Published:** 2022-10-09

**Authors:** Sabrina Lekmine, Samira Bendjedid, Ouided Benslama, Antonio Ignacio Martín-García, Samira Boussekine, Kenza Kadi, Salah Akkal, Gema Nieto, Rokayya Sami, Amina A. M. Al-Mushhin, Morooj M. Baakdah, Abeer M. Aljaadi, Saif A. Alharthy

**Affiliations:** 1 Faculty of Natural and Life Sciences, Department of Biology, Larbi Tébessi University, Tebessa 12000, Algeria; 2 Laboratory of Functional and Evolutionary Ecology, Department of Biology, Faculty of Natural Sciences and Life, Research, Chadli Bendjedid University, El Tarf 36000, Algeria; 3 Laboratory of Natural Substances, Biomolecules, and Biotechnological Applications, Department of Natural and Life Sciences, Larbi Ben M’Hidi University, Oum El Bouaghi 04000, Algeria; 4 Estación Experimental del Zaidín (CSIC) Profesor Albareda 1, 18008 Granada, Spain; 5 Biotechnology, Water, Environment and Health Laboratory, Abbes Laghrour University, Khenchela 40004, Algeria; 6 Valorization of Natural Resources, Bioactive Molecules and Biological Analysis Unit, Department of Chemistry, University of Mentouri Constantine1, Constantine 1, Constantine 25000, Algeria; 7 Department of Food Technology, Food Science and Nutrition, Faculty of Veterinary Sciences, University of Murcia, Regional Campus of International Excellence “Campus Mare Nostrum”, Espinardo, 30071 Murcia, Spain; 8 Department of Food Science and Nutrition, College of Sciences, Taif University, P.O. Box 11099, Taif 21944, Saudi Arabia; 9 Department of Biology, College of Science and Humanities in Al-Kharj, Prince Sattam Bin Abdulaziz University, Al-Kharj 11942, Saudi Arabia; 10 Department of Chemistry, Preparatory Year Program, Batterjee Medical College, Jeddah 21442, Saudi Arabia; 11 Clinical Nutrition Department, Faculty of Applied Medical Sciences, Umm Al-Qura University, P.O. Box 715, Makkah 24382, Saudi Arabia; 12 Department of Medical Laboratory Sciences, Faculty of Applied Medical Sciences, King Abdulaziz University, P.O. Box 80216, Jeddah 21589, Saudi Arabia; 13 Toxicology and Forensic Sciences Unit, King Fahd Medical Research Center, King Abdulaziz University, P.O. Box 80216, Jeddah 21589, Saudi Arabia

**Keywords:** *Astragalus armatus* Willd., ultrasound, LC–MS/MS, chlorogenic acid, rosmarinic acid, molecular docking

## Abstract

The *Astragalus armatus* Willd. plant’s phenolic constituent extraction and identification were optimized using the ultrasound-assisted extraction (UAE) method and the LC–MS/MS analysis, respectively. Additionally, cupric reducing antioxidant capacity (CUPRAC), beta carotene, reducing power, DMSO alcalin, silver nanoparticle (SNP)-based method, phenanthroline, and hydroxyl radical tests were utilized to assess the extract’s antioxidant capacity, while the neuroprotective effect was examined in vitro against acetylcholinesterase enzyme. This study accurately estimated the chemical bonding between the identified phenolic molecules derived from LC–MS/MS and the AChE. The extract was found to contain sixteen phenolic substances, and rosmarinic, protocatechuic, and chlorogenic acids, as well as 4-hydroxybenzoic, hyperoside, and hesperidin, were the most abundant substances in the extract. In all antioxidant experiments, the plant extract demonstrated strong antioxidant activity and a significant inhibitory impact against AChE (40.25 ± 1.41 μg/mL). According to molecular docking affinity to the enzyme AChE, the top-five molecules were found to be luteolin, quercetin, naringenin, rosmarinic acid, and kaempferol. Furthermore, these tested polyphenols satisfy the essential requirements for drug-like characteristics and Lipinski’s rule of five. These results highlight the significance of the *A. armatus* plant in cosmetics, as food additives, and in the pharmaceutical industry due to its rosmarinic and chlorogenic acid content.

## 1. Introduction

Synthetic compounds, notably tert-butylhydroquinone (TBHQ), propyl gallate (PG), and butylated hydroxytoluene (BHT), have been widely employed in the nutraceutical industries as the off-flavor development of foods as well as in pharmaceuticals to fight lipid oxidation [1].

Due to the negative impacts of these synthetic molecules, numerous research studies have concentrated on bioactive molecules from plants, which are used as safe antioxidant compounds [2,3]. These biomolecules have significant importance in reducing oxidative stress, which could destroy the biological mechanisms in the human body [3]. In reality, the preference for natural substances is also a consequence of the lack of adverse effects where numerous studies have shown that natural compounds counteract the negative effects of pharmaceutical treatments, implying that these natural molecules have greater medicinal value [4].

Various illnesses have been treated using plants [5] because of the existence of polyphenols, which have many biological activities [5]. Furthermore, the studies on enzyme inhibitors are regarded as the most effective in vitro strategies for a variety of diseases [6]. However, a complete understanding of the underlying physiological systems is required for the creation of therapeutic approaches [6]. Alzheimer’s disease (AD), or neurodegenerative disease, is currently affecting older adults more than ever before. AD patients’ brains were shown to have hyperactivity of acetylcholinesterase (AChE) [7]. The depletion of the acetylcholine (ACh) neurotransmitter by cholinesterase is linked to Alzheimer’s etiology [7]. Currently, the most successful therapeutic strategy is the application of ChE inhibitors [8], particularly natural anticholinesterases and antioxidants. As most of these medicines have numerous adverse effects, it is desirable to use and manage them effectively.

Legumes are typically a significant source of high-quality protein, vitamins, minerals, and bioactive compounds [9]. The growing demand for leguminous plants as a result of the scarcity of agricultural land and water resources is turning into a severe problem. Thus, food plants with substantial levels of bioactive substances are becoming more difficult to acquire and much more expensive. As a result, an important research tendency in recent years has been the discovery of additional raw and environmentally safe sources of essential nutrients, particularly from non-conventional plants [10], while still able of offering excellent nutrients for industrial use under salinity and dry situations [10].

The plants from the *Astragalus* genus, members of the *Fabaceae* family, are often utilized in food and medicine as well as feed for livestock [9,10]. Among these herbs, we are interested in *Astragalus armatus* Willd., which belongs to the *Leguminosae* plant and is prevalent in Mediterranean climate zones in Europe and North Africa [9]. This species is also found in Algeria’s Sahara Desert, Morocco, and Tunisia [10]. Aromatic compounds and essential oils are found in several *Astragalus* species, with a variety of medicinal and economic applications [11]. The capacity of this species to inhibit the enzymes associated with a variety of chronic illnesses has been demonstrated by several studies [10,11,12]. It has been demonstrated that several bioactive substances from this genus are effective in cancer treatment [12], including saponins, which had a substantial anticancer impact on hamsters [12]. Several polysaccharides and phenolic compounds with immunostimulatory properties are also found in this species [12].

However, a variety of solvents and methods have been employed to extract bioactive compounds from plants [12]. For this purpose, the current study aimed to extract the phenolic compound using an ultrasound-assisted extraction method, investigates the metabolite profiling of *A. armatus* using LC–MS/MS analyses, and assesses both its in vitro antioxidant potential and anticholinesterase effects. This study also reports a molecular docking analysis, where the identified polyphenols were used as ligands to test their inhibitory activity against AChE receptors to learn more about their mechanisms of action.

## 2. Materials and Methods

### 2.1. Chemicals and Instruments

The chemical profile was performed by using LC–MS/MS (Shimadzu, Kyoto, Japan). The activity assays were evaluated by using a Shimadzu UV spectrophotometer and a BioTek Power Wave XS microplate reader (USA). All the standard chemicals utilized in the antioxidant activities and LC–MS/MS were purchased from Merck. Germany Sigma provided the AChE as well as the reactive products of antioxidant tests (Germany). All solvents were of analytical grade [13].

### 2.2. Chemical Identification and Measurement of Phenolic Compound

#### 2.2.1. Plant Extract Preparation

The aerial parts of *A. armatus* were taken from Algeria’s Sahara Desert (EL Oued). Therefore, the ultrasound-assisted extraction method was used in this investigation to perform the plant extraction. An ethanol–water mixture (70:30 *v/v*) was used to extract the plant. Following concentration with a rotary evaporator, the residual material was dissolved in water and then extracted using petroleum ether and ethanol, respectively [14]. The ethanolic fraction is the focus of our investigation.

#### 2.2.2. Equipment and Chromatographic Parameters

The tandem MS system used in conjunction with the UHPLC (Nexera type Shimadzu, USA) was used for the LC–MS/MS technique [15]. Furthermore, The LC–MS analysis was also performed using LC-30AD binary pumps, a CTO-10ASvp column oven, a DGU-20A3R degasser, and a SIL-30AC autosampler, and the separation was carried out using a reversed-phase C18 Inertsil ODS-4 analytical column of 150 mm × 4.6 mm × 3 m at 40 °C. Mobile phase A (H_2_O, ammonium formate (5 mM), and formic acid 0.1%) and mobile phase B (methanol, ammonium formate (5 mM), and formic acid 0.1%) were used in the elution gradient. The solvent flow rate was kept at 0.5 mL/min, and the injection was fixed at 4 µL. The ESI source was used in air pressure ionization, where the optimal ESI circumstances were determined to be a DL temperature of 250 °C, an interface temperature of 350 °C, a heat block temperature of 400 °C, a drying gas flow rate of 15 L/min, and a nebulizing gas flow rate of 3 L/min.

#### 2.2.3. MS Instrumentation

The MS identification was performed using a Shimadzu LCMS 8040 triple quadrupole mass spectrometer equipped with an ESI source that could operate in both positive and negative ionization mechanisms. The data from LC–MS/MS was collected and processed using Lab Solutions software (Shimadzu, Kyoto, Japan). The multiple reaction monitoring (MRM) strategy was used to quantify the analyses. The assay of the explored substances was carried out after two or three shifts per substance. The first one was for quantification, whereas the second and/or third were for confirmation.

#### 2.2.4. Method Validation Parameters for LC–MS/MS

The detailed analytical characteristics of LC–MS/MS reference compounds have already been documented in the literature [13,14,15], including the linearity ranges and rectilinear regression estimates for the standard chemicals examined. All the calibration curves for all chemical compounds were linear and reproducible, with a correlation coefficient of more than 0.991. Ertas and Yener [13,14,15] depict, furthermore, the disclosed analytical method’s limit of detection (LOD) and limit of quantitation (LOQ). LOD varied from 0.05 to 25.8 g/L, while LOQ varied from 0.17 to 85.9 g/L. Furthermore, phenolic compound recoveries ranged from 96.9% to 106.2%.

### 2.3. Antioxidant Activity

The details of all biological activity studies are provided as Supplementary Material. A superoxide alkaline DMSO test, reducing power test, βeta-carotene bleaching test, cupric reducing assay (CUPRAC) measuring antioxidant capacity, the test of scavenging of hydroxyl radicals, o-phenanthroline assay, and the silver-nanoparticle-based method were used to estimate the antioxidant potential [15,16,17,18,19,20,21,22].

### 2.4. Acetylcholinesterase Inhibitory Assay

The anti-AChE inhibitory assay of *A. armatus* extract was performed using the technique outlined in Ellman’s work [23]. Briefly, 150 μL of a sodium phosphate buffer (100 Mm, pH 8.0), 10 μL of the extract, and a volume of 20 μL AChE were mixed. Then, 15 min after incubation at a suitable temperature for enzyme activation, 10 μL DTNB (0.5 mM) was introduced to the reactive mix. After that, 20 L of acetylthiocholine iodide was added to begin the reaction (0.71 mM). A 96-well microplate reader was used to measure the absorbance, and the percent inhibition was calculated as follows:

(E − S)/(E) × 100

where E is the enzyme’s activity without the extracts, and S is the enzyme’s activity with the extracts.

The experiments were carried out in triplicate. The standard compound used in the experiments was galantamine. A 50% inhibitory concentration was taken to represent the results (IC_50_).

### 2.5. Docking Molecular Analysis

This study used docking molecular analysis to measure the binding degree affinity between AChE and the polyphenols of LC–MS/MS results. First, the crystal structure of the AChE enzyme with ID 4EY6 was loaded from the PDB database (https://www.rcsb.org, accessed on 23 August 2022). The 3D structure of this receptor was prepared using the Chimera 1.15 program by removing the ligands and the co-crystallized solvents, as well as the water molecules. The global atomic charge of the protein was also corrected, and the H-atoms were added. On the other hand, the structures of the polyphenols of LC–MS/MS serving as ligands were obtained from the PubChem database (https://pubchem.ncbi.nlm.nih.gov/, accessed on 23 August 2022) in their SMILES formats, where they were converted into 3D structures using the Chimera package, which was also used to assign them Gasteiger charges and hydrogen atoms. The program AutoDock Vina was used to perform molecular docking. The outcomes were expressed in terms of binding affinity, where the ligands with the highest binding affinity were selected for the investigation of their interactions with the AChE active site.

### 2.6. Drug Likeness and ADMET Profiling

The drug-like qualities of the chosen phenolic substances were measured with the Swiss ADME Web Service and using the rules of Lipinski and Veber [24,25], the different biochemical attributes were determined. In addition, the two web applications ProTox-II (https://tox-new.charite.de, accessed on 26 August 2022) [26] and ADMETlab 2.0 (https://admetmesh.scbdd.com/, accessed on 26 August 2022) [27] were used to predict the toxicity of the selected ligands.

### 2.7. Statistical Analysis

The measurements were carried out in triplicate for each sample. The findings are expressed as mean ±standard deviation (SD). A one-way analysis of variance (ANOVA) of the bioassays was conducted using the PRISM GraphPad V: 5.00 (Trial), followed by the Tukey test at *p* 0.05.

## 3. Results and Discussion

### 3.1. Chemical Identification and Measurement of Phenolic Compounds

Several plants ingested as food have attracted increased interest due to the preventive impact of their phenolic compounds on cellular oxidative damage [13,14] and their availability in the human diet [28].

**Figure 1:**
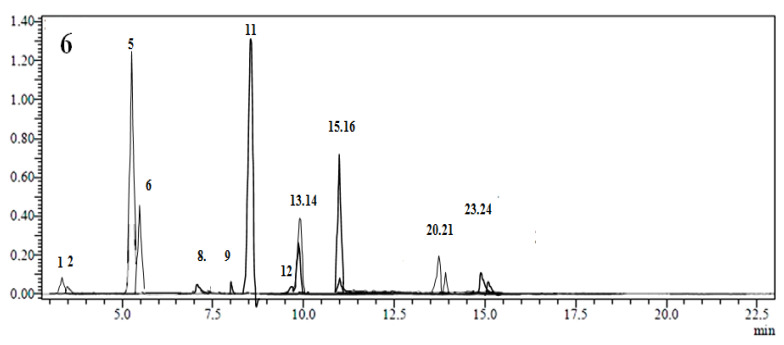
Chromatogram of LC–MS/MS-analyzed *A. armatus* extract.

Numerous studies have been conducted on LC–MS/MS utilization for sensitive biomolecule characterization in plant extracts [14,15,16,17,18,19,20,21,22,23,24,25,26,27,28]. As a result, the developed method of a triple quadrupole detector was used in this study. This method is considered more effective than other LC methods due to its high selectivity and sensitivity [5]. The base peak chromatogram (BPC) of *A. armatus* extract is presented in Figure 1, while the quantitative results are presented in Table 1.

**Figure 2:**
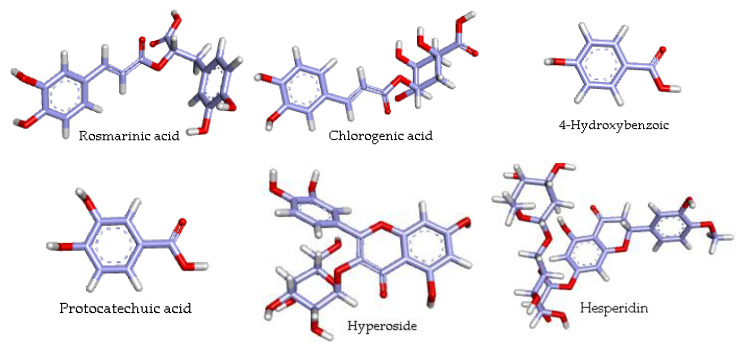
The 3D structures of the most abundant compounds in *A. armatus* extract visualized using Discovery Studio.

In total, 16 molecules were identified and quantified in the tested plant. The differences were highly significant between the concentrations of phenolic compounds contained in the ethanolic extract (*p* < 0.05). It was clear that the major identified phenolic compounds were rosmarinic acid, chlorogenic acid, 4-hydroxybenzoic, protocatechuic acid, hyperoside, and hesperidin. Their 3D structures are presented in Figure 2. There was no significant variation in rosmarinic and chlorogenic acid concentrations. (*p* < 0.05).

In terms of the flavonoids, it was also remarkable to find a considerable level of quercetin (21.9 ± 2.1 µg/g), naringenin (5.3 ± 0.3 µg/g), luteolin (29.5 ± 2.0 µg/g), and kaempferol (35.0 ± 2.3 µg/g) in the plant extract. Additionally, a moderate amount of rutin (79.3 ± 22.7 µg/g), vanillin (65.3 ± 3.6 µg/g), salicylic acid (27.2 ± 1.4 µg/g), and low levels of tr-caffeic acid (9.8 ± 0.8 µg/g) were also observed.

In terms of non-phenolic compounds, *A. armatus* was found to contain a significant amount of quinic acid (12214.7 ± 664.3 µg/g) and a low value of malic acid (30.6 ± 2.3 µg/g) (Figure 1 and Table 1). Among the 27 standard chemicals used, gallic acid, tr-acotinic acid, myricetin, tannic acid, rhamnetin, coumarin, apigenin, chrysin, and fisetin were not found in the *A. armatus* extract.

Similar to the present work, and by using LC–ESI–MS, it has been documented that the *A. armatus* ethanolic fraction contains quercetin and kaempferol in low concentrations [12]. It was also mentioned that the *A. armatus* ethanolic fraction produced through sample extraction contained significant levels of cirsiliol (37.1 ± 1 µg/g DM).

The high concentration of phenolic content in the *Astragalus* genus has been observed previously [29]. Their structures were elucidated by the nuclear magnetic resonance (NMR) technical analysis in the form of 4′-dimethoxy isoflavane, 7,2′-dihydroxy-3′, isoliquiritigenin, formononetin, quercetin, ononin, kaempferol, vanillic, and p-hydroxybenzoic acids [29]. In addition, luteolin and its glucoside have been also isolated and described from *A. galegiformis* L., which confirms the richness of this genus in phenolic compounds. These advantageous compounds found in the extract explain the effectiveness of the ultrasound-assisted extraction technique on the determination of phenolic concentrations [1].

Additionally, this study provided the first significant findings on the high concentration of rosmarinic and chlorogenic acids in *A. armatus*. It may be suggested that this plant is a potential source of these two major substances. Several plant species have reportedly been tested to lengthen the shelf life of cheese and increase its quality, as well as for potential application in bread and cupcakes because of their high rosmarinic acid concentration [30]. Our results reinforce the possibility of also using this plant in this commercial context.

### 3.2. Antioxidant Activity

The search for bio-antioxidants with potential applications in the food, cosmetics, and medical sectors is now gaining more interest among researchers [5]. The antioxidant activity of plant extracts has an important impact on a variety of processes, including their ability to reduce and scavenge free radicals as well as their capacity to absorb oxygen radicals [5]. As a consequence, the antioxidant potential of the ethanolic extract of *A. armatus* was estimated using seven antioxidant tests (Table 2).

To our knowledge, there is no published research in the literature on *A. armatus* using these antioxidant techniques. As shown in Table 2, the terms “IC_50_” and “A0.50 levels” refer to the concentration at 0.50 absorbance and the 50% inhibitory percent quantity, respectively. These two values were calculated using a linear regression approach and are presented as mean SD (n = 3). The ethanolic extract was found to have a significant antioxidant activity, drawing more attention to this leguminous plant as a very promising natural antioxidant that could potentially be engaged in the treatment of various physiological pathologies caused by imbalances in oxidative systems. The observed variances in antioxidant effects that are influenced by the extracting solvent utilized have previously been examined [5]. For this reason, we can confirm that the method of extraction can significantly contributes to increasing the yield of polyphenolic content and antioxidant capacity.

Additionally, antioxidants are very essential for preventing lipid peroxidation and cellular damage caused by free radicals [28]. Lipid peroxidation is a chain reaction involving free radicals, which are associated with a wide range of biological diseases [31]. As indicated by the results, the *A. armatus* extract demonstrated a potent anti-lipid peroxidation activity with a high half-maximal inhibitory concentration of 5.12 ±1.2 µg/mL more than α-Tocopherol and BHT (11.43 ± 0.23 and 9.65 ± 1.1 µg/mL), which served as the standards for the β-Carotene bleaching method. As shown in Table 2, rosmarinic and chlorogenic acids are both present in very high concentrations in the *A. armatus* extract (80,695.32 and 76,635.0 µg/g dry extract, respectively). Therefore, it may be suggested that the observed activity is caused by rosmarinic and chlorogenic acids and their synergic effects with other phenolic compounds.

### 3.3. Acetylcholinesterase Inhibitory Assay

Plants are the most important source to create novel AChE inhibitor drugs for the treatment of degenerative disorders, including AD, which is considered the most common disease worldwide.

A variety of endogenous enzymes must be inhibited in order to reduce this neuropathology [32]. There have been reports that a variety of plant compounds in extracts are effective alternatives for treating Alzheimer’s disease. These bioactive substances have potent antioxidant activity that combats free radicals and reduces brain cell destruction [33,34].

There are currently few studies on the *Astragalus* genus and the ability of its species to inhibit enzyme function. Through this research, we aimed to demonstrate this plant’s capacity to obstruct AChE’s catalytic site.

As seen in Table 3, the AChE inhibitory activity of *A. armatus* was compared with that of galantamine. The plant’s IC_50_ value for blocking AChE was 40.25 µg/mL. The analysis using statistics demonstrates that the values were significantly different (*p* < 0.05).

These findings can be explained by the existence of chemicals in the extracts that are responsible for the inhibition of the AChE [4] and particularly by the ethanolic extract’s flavonoids. Our results agree with those of the investigations conducted by Teyeb et al. [35]. Additionally, the *A. setulosus* plant exhibited a good inhibition against BChE, according to a previous study by Zengin [36], while *A. leporinus* Boiss. var. hirsutus extract demonstrated a potent inhibitory activity, with an IC_50_ value of 66.15 ± 4.08 µg/mL.

### 3.4. Binding Mode Analysis Using a Molecular Docking Approach

Molecular docking has become a much more frequently used technique in the computer-aided drug development process. This groundbreaking strategy can significantly reduce energy use, costs, and time in drug discovery by screening large pharmaceutical libraries for future drug substances [37]. In the current work, we used a molecular docking method to screen the inhibitory effects of the polyphenolic components of LC–MS/MS.

It is well-established from crystal studies that Ser203 and His447 are the two major amino acids important for AChE’s catalytic properties. These two are located in the catalytic and oxyanion hole residues, which also include Glu202, Tyr33, and Trp86, located in the choline-binding pocket, and Trp236, Phe338, Phe297, and Phe295, composing the acyl-binding pocket of the enzyme [38,39]. The binding mode of the co-crystallized inhibitor galantamine, as shown in Table 4, clearly identified these key residues.

**Figure 3:**
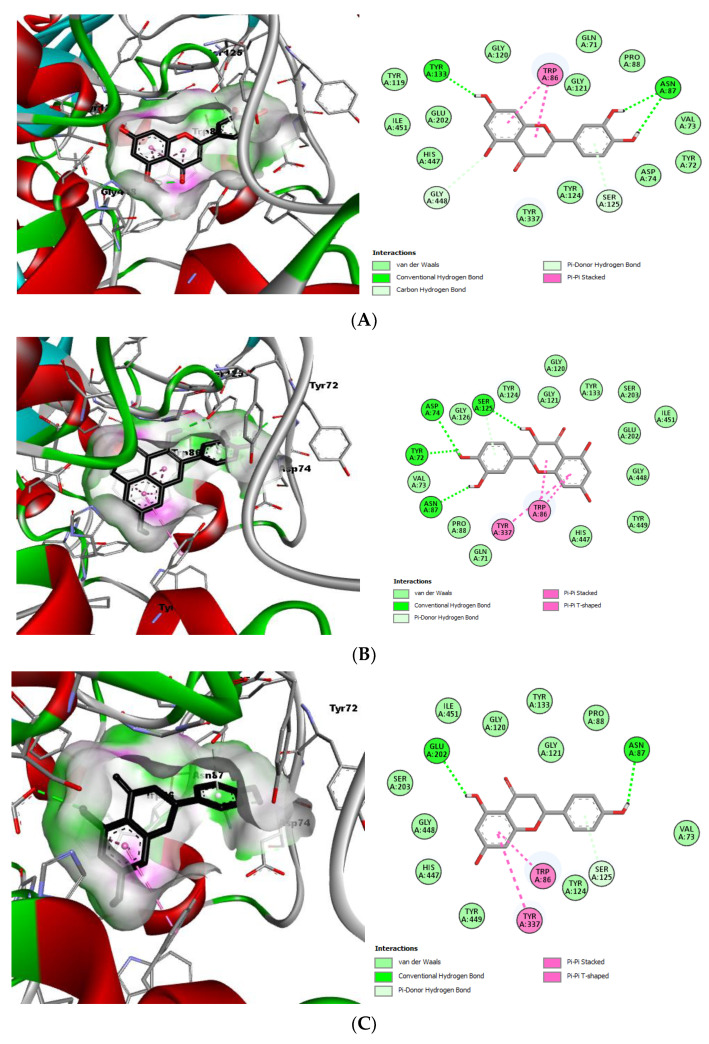

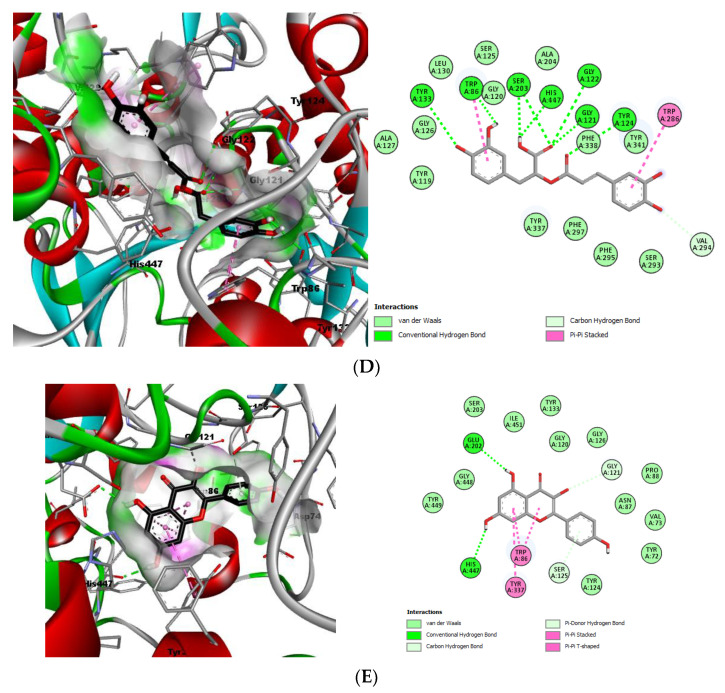
Predictions of the top-five ligands’ binding modes with AChE (4EY6). Profile view and 2D schematic diagrams of (**A**) luteolin, (**B**) quercetin, (**C**) naringenin, (**D**) rosmarinic acid, and (**E**) kaempferol, trapped in AChE’s gorge pocket.

The docked reference molecule (galantamine) gave a binding energy value of −10.3 Kcal/mol and showed four hydrogen bonds, including one with the key amino acid Glu202. In addition, this molecule interacts by forming a P-Stacked amide bond with Gly121 and six Pi–alkyl bonds with Tyr337, Phe338, Phe295, Phe297, His447, and Trp86. Based on the high scores given by the top-five compounds (Table 4), it is evident that they all had a very strong affinity with the catalytic site of the enzyme. In addition, they all had interactions with at least one of the key amino acids (Table 4). With a docked score of −10.8 Kcal/mol, luteolin was determined as the ligand with the greatest inhibition potential. This molecule interacts by forming five hydrogen bonds with Asn87, Tyr133, Gly448, and Ser125. The key amino acid Trp86 provides two Pi–Pi-stacked interactions with these ligands. Moreover, several Van der Waals attractions reinforce the stability of the luteolin–AChE complex (Figure 3).

### 3.5. Drug-Likeness and ADMET Profiling

In Table 5, the ADME results of the top-five ligands measured using the SWISS-ADME server are displayed. Luteolin, quercetin, naringenin, rosmarinic acid, and kaempferol have molecular weights of 286.24, 302.24, 272.25, 360.31, and 286.24 g/mol, respectively; the results of these phenolic substances suggested that they could be easily transported, distributed, and absorbed through the biological membranes [25,26,27,28,29,30,31,32,33,34,35,36,37,38,39,40]. In addition, luteolin, quercetin, naringenin, rosmarinic acid, and kaempferol were found to have LogP values of 1.73, 1.23, 1.84, 1.52, and 1.58, respectively, which are consistent with Lipinski’s rule of five. Additionally, the hydrogen bond donor and acceptor numbers of these ligands were less than five and less than ten, respectively, which satisfies the ADME standards of H-bond donors and acceptors. The ADME results showed that luteolin, quercetin, naringenin, rosmarinic acid, and kaempferol had topological polar surface (TPSA) values of 111.13, 131.36, 86.99, 144.52, and 111.13 Å2, respectively. These values fall within the acceptable range not exceeding Å2, as outlined earlier by Cecchelli [41]. Furthermore, the atom molar refractivity (AMR) values of the selected components varied between 71.57 for naringenin and 91.40 for rosmarinic acid, both of which fall within the margins designated for this criterion between 40 and 130 [42]. Compound solubility is another important biochemical criterion for the choice of a biologically active molecule. LogS is the measurement that represents this parameter. For the five best polyphenolic compounds, this measurement was between −3.71 for luteolin and −3.16 for quercetin, reflecting good solubility, which corresponds to fairly good absorption and distribution. The drug-likeness analysis of the top-five ligands of LC–MS/MS revealed that these metabolites have an advantageous pharmacological profile and can thus be classified as drug-like substances. The toxic behavior of the five ligands was also studied, and its results are reported in Table 6. The results from this analysis are more or less reassuring of the safety of these molecules. None of the ligands are hepatotoxic or androgenic disruptors. However, quercetin showed potential for carcinogenic and mutagenic activity, and similar to galantamine, chlorogenic acid may pose an immunotoxicological risk.

## 4. Conclusions

The findings of this study provide the first details on the effects of the ultrasound-assisted method of extraction on the chemical profiles as well as the antioxidants of the *A. armatus* extract collected from EL Oued, Algeria. As a consequence, the analysis of phytochemical constituents using the LC–MS/MS instrument attested to the presence of rosmarinic and cholinergic acids, with a high amount in the ethanolic fraction of *A. armatus*. Our findings also provide additional information to the literature data about the high antioxidant potential of the *A. armatus* plant by analyzing the results of reducing power, CUPRAC, beta carotene, DMSO alcalin, silver nanoparticle (SNP)-based method, phenanthroline, and hydroxyl radicals.

The present comprehensive study contends that five phytoconstituents, i.e., luteolin, quercetin, naringenin, rosmarinic acid, and kaempferol, are promising AChE inhibitors. Integrated molecular docking disclosed that these polyphenols establish a stable structure with the enzyme AChE with strong affinities. Furthermore, their potential inhibitors adhere to the requirements for drug-likeness and ADME assets, relying on Lipinski’s rule of five. As a result, the *A. armatus* plant can be used in food additives and preservatives due to its high concentration of chlorogenic and rosmarinic acids, as well as to replace synthetic antioxidants in cosmetic and pharmaceutical products.

## Figures and Tables

**Table 1:** LC–MS/MS data of polyphenols in *A. armatus* extract.

Peak and Molecules		Rt Retention Time	MS2 (Collision Energy)	Quantification (µg Analyte/g Extract)
				*A. armatus*
1	Quinic acid	3.32	85 (22), 93 (22)	963.12 ± 22.3 g
2	Malic acid	3.54	115 (14), 71 (17)	30.6 ± 2.3 i
3	tr-Aconitic acid	4.13	85 (12), 129 (9)	/
4	Gallic acid	4.29	125 (14), 79 (25)	/
5	Chlorogenic acid	5.43	191 (17)	76,635.0 ± 6.2 a
6	Protocatechuic acid	5.63	109 (16), 108 (26)	43,986.0 ± 1.2 b
7	Tannic acid	6.46	124 (22), 78 (34)	/
8	tr-Caffeic acid	7.37	135 (15), 134 (24), 89 (31)	86.71 ± 1.3 i
9	Vanillin	8.77	136 (17), 92 (21)	456.7 ± 2.4 h
10	p-Coumaric acid	9.53	119 (15), 93 (31)	/
11	Rosmarinic acid	9.57	161 (17), 133 (42)	80,695.32 ± 12.3 a
12	Rutin	10.18	300 (37), 271 (51), 301 (38)	856.5 ± 1.6 g
13	Hesperidin	9.69	303, 465	19,676.0 ± 3.2 c
14	Hyperoside	10.43	300, 301	23,976.0 ± 1.8 c
15	4-OH Benzoic acid	11.72	93, 65	59,986.0 ± 6.3 b
16	Salicylic acid	11.72	93, 65, 75	433.71 ± 3.3 h
17	Myricetin	11.94	179, 151, 137	/
18	Fisetin	12.61	135, 121	/
19	Coumarin	12.52	103, 91, 77	/
20	Quercetin	14.48	179, 151, 121	6253.3 ± 2.1 d
21	Naringenin	14.66	151, 119, 107	4254.7 ± 3.1 de
22	Hesperetin	15.29	164, 136, 108	/
23	Luteolin	15.43	175, 151, 133	3221.5 ± 6.3 e
24	Kaempferol	15.43	217, 133, 151	2451.5 ± 1.8 ef
25	Apigenin	17.31	151, 117	/
26	Rhamnetin	18.94	165, 121, 300	/
27	Chrysin	21.18	143, 119, 107	/

/: Not found. MS^2^: segments of the MRM for the associated molecular ions. Significant differences exist between the values with various letters (a, b, c, d, e, f, g, h, and i) (*p* 0.05).

**Table 2:** Antioxidant activity the ethanolic fraction of *A. armatus*.

Products	CUPRAC (A0.5)	Reducing Power (A0.5)	Beta Carotene (IC_50_)	DMSO Alcalin (IC_50_)	SNP (IC_50_)	Phenanthroline (A0.5)	Hydroxyl Radical (IC_50_)
*A. armatus*	14.58 ± 4.56 c	25 ± 1.12 b	5.12 ±1.2 a	13 ± 1.25 b	10.2 ± 1.7 b	66 ± 1.14 d	25 ± 1.2 b
BHT *	8.86 ± 2.8 b	/	9.65 ± 1.1 b	/	/	2.33 ± 1.7 b	/
BHA *	5.26 ± 1.6 a	/	9.82 ± 2.1 b	/	/	2.84 ± 2.7 b	/
A-tocopherol *	/	34.93 ± 2.38 c	11.43 ± 0.23 c	4.2 ± 0.95 a	/	/	/
Ascorbic acid *	8.31 ± 0.1 b	6.77 ± 1.15 a	/	/	7.14 ± 0.05 a	3.08 ± 0.02 a	12.33 ± 1.17 a
Tannic acid *	/	5.17 ± 1.2 a	/	3.3 ± 0.91 a	/	/	/
Trolox *	8.69 ± 0.1 b	5.23 ± 1.2 a	/	/	33.26 ± 2.1 c	5.21 ± 0.27 c	/

* Reference compounds. /, not tested.

**Table 3:** Galantamine standard and *A. armatus* fraction IC_50_ values (g/mL) for inhibiting acetylcholinesterase activity.

Extract	IC50 Values (g/mL) for Inhibiting Acetylcholinesterase Activity
*A. armatus*	40.25 ± 1.3 ^a^
Galantamine	34.75 ± 1.1 ^b^

**Table 4:** The best results for the docking of LC–MS/MS polyphenolic ligands with AChE target.

	Binding Energy (Kcal/mol)	Hydrogen Interactions	Hydrophobic Interactions	Van der Waals Interactions
Galantamine	−10.3	Ser203, Glu202, Tyr124, Asp74, His447	Tyr337, Gly121, Phe338, Phe295, Phe297, His447, Trp86	Gly122, Ser125, Tyr341, Gly120, Tyr133, Gly448
Luteolin	−10.8	Asn87, Tyr133, Gly448, Ser125	Trp86	Pro88, Gln71, Gly121, Gly120, Tyr199, Glu202, Ile451, His447, Tyr337, Tyr124, Asp74, Tyr72, Val73
Quercetin	−10.6	Ser125, Asp74, Tyr72, Asn87	Trp86, Tyr337	Ile451, His447, Tyr449, Gly448, Glu202, Ser203, Tyr133, Gly120, Gly121, Tyr124, Gly126, Val73, Pro88, Gln71
Naringenin	−10.2	Ser125, Asn87, Glu202	Trp86, Tyr337	Val73, Tyr124, Tyr449, His447, Gly448, Ser203, Ile451, Gly120, Tyr133, Gly121, Pro88, Gly121
Rosmarinic acid	−10.2	Tyr124, Gly121, Gly122, His447, Ser203, Trp86, Tyr133, Val294	Trp86, Trp286	Ser293, Phe295, Phe297, Tyr337, Tyr119, Ala127, Gly126, Leu130, Ser125, Gly120, ALA204, Phe338, Tyr341
Kaempferol	−10.0	Glu202, His447, Gly121	Trp86, Tyr337	Tyr124, Tyr72, Val73, Asn87, Pro88, Gly126, Gly120, Tyr133, Ile451, Ser203, Gly448, Tyr449

**Table 5:** Drug-like properties of the top active ligands of LC–MS/MS.

	MW g/mol	LogP	LogS	HBA	HBD	TPSA (Å^2^)	AMR	nRB	Lipinski	Veber
Galantamine	287.35	1.91	−2.93	1	4	41.93	84.05	1	Yes	Yes
Luteolin	286.24	1.73	−3.71	6	4	111.13	76.01	1	Yes	Yes
Quercetin	302.24	1.23	−3.16	7	5	131.36	78.03	1	Yes	Yes
Naringenin	272.25	1.84	−3.49	5	3	86.99	71.57	1	Yes	Yes
Rosmarinic acid	360.31	1.52	−3.44	8	5	144.52	91.40	7	Yes	No
Kaempferol	286.24	1.58	−3.31	6	4	111.13	76.01	1	Yes	Yes

HBA, Num. H-bond acceptors; HBD, Num. H-bond donors; nRB, Num. rotatable bonds; AMR, atom molar refractivity.

**Table 6:** Drug-like properties of the top active ligands of LC–MS/MS.

	Criteria	Galantamine	Luteolin	Quercetin	Naringenin	Chlorogenic Acid	Kaempferol
Absorption-Distribution	BBB penetration	Yes	No	No	No	No	No
	Caco2	High	High	Low	High	Low	High
	HIA	High	High	High	High	Low	High
Metabolism	CYP1A2 inhibitor	No	yes	Yes	No	No	Yes
	CYP2C19 inhibitor	No	No	No	No	No	No
	CYP2C9 inhibitor	No	No	No	No	No	No
	CYP2D6 inhibitor	Yes	Yes	Yes	Yes	No	Yes
	CYP3A4 inhibitor	No	Yes	Yes	Yes	No	Yes
Excretion	Cl	Low	Moderate	Moderate	High	Low	Moderate
Toxicity	hERG Blockers	No	No	No	No	No	No
	AMES Toxicity	No	Yes	Yes	No	No	No
	Carcinogenicity	No	No	Yes	No	No	No
	Cytotoxicity	No	No	No	Yes	No	No
	Immunotoxicity	Yes	No	No	No	Yes	No
	H-HT	No	No	No	No	No	No
	NR-AR	No	No	No	No	No	No
	NR-ER	No	Yes	Yes	Yes	No	Yes
	SR-p53	No	No	No	Yes	No	No

BBB, Blood–brain barrier; HIA, human intestinal absorption; Caco2, permeability assay; hERG, human ether-a-go-go-related gene potassium channel; H-HT, human hepatotoxicity; NR-AR, androgen receptor disruptor; NR-ER, estrogen receptor disruptor; SR-p53, tumor suppressor protein p53 activator; Cl, clearance of the molecule; No, inactive; Yes, active.

## Data Availability

Data are available upon request from the corresponding author.

## Supplementary Materials

The following supporting information can be downloaded at: https://www.mdpi.com/article/10.3390/antiox11102000/s1. In our case the supplementary material is neither a table, nor a figure or video. it is rather a part of the material and method including the detailed protocols of the different techniques used to evaluate the antioxidant activity of the extract studied.
