# Supplementary material for: Ultrasound-Assisted Extraction, LC–MS/MS Analysis, Anticholinesterase, and Antioxidant Activities of Valuable Natural Metabolites from Astragalus armatus Willd.: In Silico Molecular Docking and In Vitro Enzymatic Studies
Source: Antioxidants (Basel). 2022 Oct 9;11(10):2000. doi: 10.3390/antiox11102000 (PMC9598503; doi:10.3390/antiox11102000)
Supplement: Supplementary file 1 [file antioxidants-11-02000-s001.zip › antioxidants-1950170-supplementary.pdf]

**Superoxide Alkaline DMSO Test**

The superoxide radical test was evaluated as described by Kunchandy's method. 40 μL of each sample were added to 30 μL of NBT (nitroblue tetrazolium) (1 mg/mL) and 130 μL of alkaline DMSO (1.0 mL DMSO, 5 mMNaOH, 100 μL H2O). The absorbance was measured at 560 nm, and the findings were presented as IC50 values (Kunchandy and Rao 1990).

**Reducing Power Test**

To investigate the reducing power effect, 10 μL of each extract were mixed with 40 μL of 0.2 M phosphate buffer (pH 6.6) and 50 μL of potassium ferricyanide (1%) and incubated at 50°C for 20 minutes. After that, 50 μL of TCA (trichloroacetic acid) (10%) and 10 μL of ferric chloride (0.1%) were added, and the mixture's absorbance was measured at 700 nm (Oyaizu 1986).

**β-Carotene/Linoleic Acid Bleaching Test**

The β-carotene bleaching activity of extract was evaluated Following Marco's instructions (Marco 1968), with several modifications. 0.5 mg of β-carotene dissolved in 1mL of chloroform was added to 25 $\mu$L of linoleic acid and 200 mg of Tween 40 to obtain an emulsifying mixture. After evaporating the chloroform, 100 mL of distilled water saturated with oxygen was added with strong agitation. The absorbance of the β-carotene solution should be between 0.8 and 0.9 nm. A volume of 160 μL of this prepared solution was added to 40 μL of extract at the different concentrations. Absorbance was measured at 470 nm using a microplate reader. BHA and $\alpha$-tocopherol were used as standards.

**Cupric Reducing Antioxidant Capacity (CUPRAC) Assay**

The reduction of copper was determined by the CUPRAC method described by Apak et al. 2004. In brief, the solutions tests were prepared by mixing 50 $\mu$L Cu (II) (10 mM), 50 μL neocuprine (7.5 mM), and 60 μL of NH4Ac buffer solution (1 M, pH = 7.0). To produce a final volume of 200 μL in each well of the microplate, different concentrations of extracts were added to the initial mixture. the absorbance was measured at 450 nm After 1 hour. The results were calculated as A0.5 (μg/mL) corresponding to the concentration indicating 0.50 absorbance and the reducing capacity compared to those of $\alpha$-tocopherol and BHT.

**Hydroxyl Radical Scavenging Assay**

The modified Smirnoff and Cumbes approach was used to assess the Hydroxyl Radical Scavenging Assay. 40 μL of each sample was mixed with 80 μL salicylic acid (3 mM), 24 μL FeSO4 (8 mM), and 20 μL H2O2 (20 mM). The microplate was incubated at 37 °C for 30 min. After that, 36 μL H2O was added and the absorbance was measured at 510 nm. The result was calculated given as an IC50 value (Smirnoff and Cumbes 1989).

**O-Phenanthroline Assay**

The method described by Szydlowska-Czerniaka et al. [Szydłowska-Czerniak et al., 2008] was used with no modifications. 10 μL of each extract was mixed with 30 μL o-phenanthroline (0.5% in methanol), 50 μL FeCl3 (0.2%), 110 μL methanol. After the incubation at 30 °C, the absorbance was measured at 510 nm. The result was given as an A0.5 value.

**Silver Nanoparticle-Based Method**

The principle of this method is to reduce the Ag+ to spherical silver nanoparticles (SNP) (Ӧzyurek et al. 2012). The mixture containing 130 μL of SNP solution, 50 μL of H2O, and 20 μL of the extract was incubated at 25 °C for 30 min, by using the microplc reader, the absorbance was measured at 423 nm and the results were given as A0.5 value.
